# Parameter Screening and Optimization for a Polycaprolactone-Based GTR/GBR Membrane Using Taguchi Design

**DOI:** 10.3390/ijms23158149

**Published:** 2022-07-24

**Authors:** Lohitha Kalluri, Yuanyuan Duan

**Affiliations:** Department of Biomedical Materials Science, University of Mississippi Medical Center, Jackson, MS 39216, USA; lkalluri@umc.edu

**Keywords:** eggshell membrane, electrospinning, guided tissue regeneration/guided bone regeneration, nanofibers, polycaprolactone, Taguchi orthogonal arrays

## Abstract

Our objective was to determine and optimize the significant parameters affecting mechanical properties and mean fiber diameter (MFD) of a novel GTR/GBR membrane composed of polycaprolactone (PCL) and chicken eggshell membrane (ESM). For this, we prepared electrospun membrane specimens (*n* = 16) with varying concentrations of PCL, ESM, nano-hydroxyapatite (HAp), and altered electrospinning parameters as generated by DOE++ software. After the determination of MFD and mechanical properties for all specimens, Taguchi orthogonal array L8 design was used to screen significant factors affecting the MFD and mechanical properties. PCL wt%, ESM wt%, HAp wt%, applied voltage (AV), flow rate (FR), and spinneret-collector distance (SCD) were the independent variables investigated. The response variables analyzed were MFD, tensile strength (TS), and elastic modulus. ANOVA outlined ESM wt%, HAp wt%, AV, FR, SCD, and an interactive effect between PCL wt% and AV to be the significant factors affecting modulus values of an electrospun PCL/ESM membrane (*p* < 0.05). Furthermore, concentrations of PCL and ESM were the significant factors affecting MFD (*p* < 0.05) and there were no significant factors affecting the TS values. Optimization using DOE++ software predicted that the maximal TS of 3.125 MPa, modulus of 278.168 MPa, and MFD of 882.75 nm could be achieved.

## 1. Introduction

Guided tissue regeneration/guided bone regeneration (GTR/GBR) is a surgical regenerative approach that is widely employed in clinical practice for the treatment of periodontitis. Briefly, it involves the mucogingival flap elevation around affected teeth, followed by the scaling and root planing procedures, and temporary positioning of a barrier membrane beneath the gingiva [1,2]. The biological basis of this approach is to prevent the apical migration of gingival epithelium to the space over the denuded root surface by using a barrier membrane; thus, facilitating the formation of PDL tissues and alveolar bone by PDL cells and osteoblasts, respectively [2,3,4]. The majority of commercially available resorbable GTR/GBR membranes, which is a key component of the GTR/GBR technique, are either polyester-based products (synthetic) or tissue-derived collagen-based products (natural). The polyester-based synthetic membranes are biodegradable, allow tissue integration, and are easier to handle surgically compared with non-resorbable membranes. However, their poor cell response is a major limitation [4,5]. In contrast, collagen-based membranes have shown favorable regenerative results due to their excellent cell affinity and biocompatibility. Nevertheless, they demonstrated relatively poor mechanical properties and dimensional stability due to their rapid degradation and early collapse. Additionally, the high cost, availability, poor surgical handling abilities, and potential risk of disease transmission from animal-based products limit their widespread clinical usage [3,4,5,6,7]. Thus, there is a need to develop a novel economical GTR/GBR membrane that could assimilate the advantages of natural and synthetic biomaterials and promote effective bone regeneration even in cases of moderate and severe periodontitis.

Natural chicken eggshell membrane (ESM) is a bilayered microporous structure that lies in between eggshell and egg white. It is composed of highly cross-linked interwoven protein fibers made up of collagen (Types I, V, and X), osteopontin, and sialoprotein, and is functionally equivalent to the extracellular matrix in avian egg development. It acts as a natural scaffold for biomineralization during the formation of an eggshell [8,9]. Additionally, eggshell biomineralization occurs within 24 h and is the most rapid biomineralization process ever known [9]. ESM has a unique fibrous structure on the outer and inner surfaces, which facilitates the mineralization of eggshells on the outer side while preventing the mineralization of egg yolk on the inner side. Owing to this unique barrier membrane property coupled with the rapid biomineralization process, it has gained attention as a potential natural biomaterial in GTR/GBR membrane applications [10].

Inspired by the unique natural bio-membrane-like structure and rapid biomineralization process assisted by the ESM, coupled with the enormous clinical demand for GTR/GBR membrane, we are developing this novel cost-efficient ESM-based fibrous GTR/GBR membrane using a blend electrospinning process. It is a promising, environment-friendly, and cost-effective alternative for commercial collagen-based GTR/GBR membrane products because ESM comes from abundant industrial waste, unlike collagen. However, pure ESM is a low molecular weight biodegradable polymer with poor mechanical properties, which is the main limitation for GTR/GBR membrane application. Thus, usually, it is used with another polymer either as a blend or by crosslinking or physical entrapment, etc., for potential biomedical applications [10,11,12,13,14,15,16,17,18,19,20,21]. In this study, we blend electrospun ESM with Poly (ε-caprolactone) (PCL), a synthetic biopolymer, and a bioceramic nano-hydroxyapatite (HAp) to prepare a novel composite GTR/GBR membrane.

An ideal GTR/GBR membrane should fulfill all the desirable characteristics such as mechanical properties including tensile strength (TS) and modulus, porosity, biocompatibility, and controlled biodegradation [2]. Thus, the membrane composition should be optimized to achieve a compromise between the minimal requirements, target levels, and relative importance of desired mechanical properties and mean fiber diameter (MFD). Apart from the membrane composition (ESM wt%, PCL wt%, HAp wt%), other factors such as electrospinning parameters (flow rate of solution (FR), spinneret-collector distance (SCD), and applied voltage (AV)) also might have a significant effect on the physical, mechanical, and biological properties of this novel electrospun membrane. Thus, the Design of Experiments (DOE++) approach using Taguchi orthogonal array design is an effective tool to outline the significant factors and to further optimize the membrane composition to achieve the desired outcomes.

Thus, the objectives of this study were to determine and optimize the significant factors affecting the mechanical properties as well as the MFD of a novel biomimetic electrospun composite GTR/GBR membrane, using Taguchi orthogonal arrays in DOE++ software. The null hypothesis was that there was no significant difference in TS, modulus, and MFD of PCL/ESM composite membrane with electrospinning parameters or the polymer solution composition.

## 2. Results

**Figure 1:**
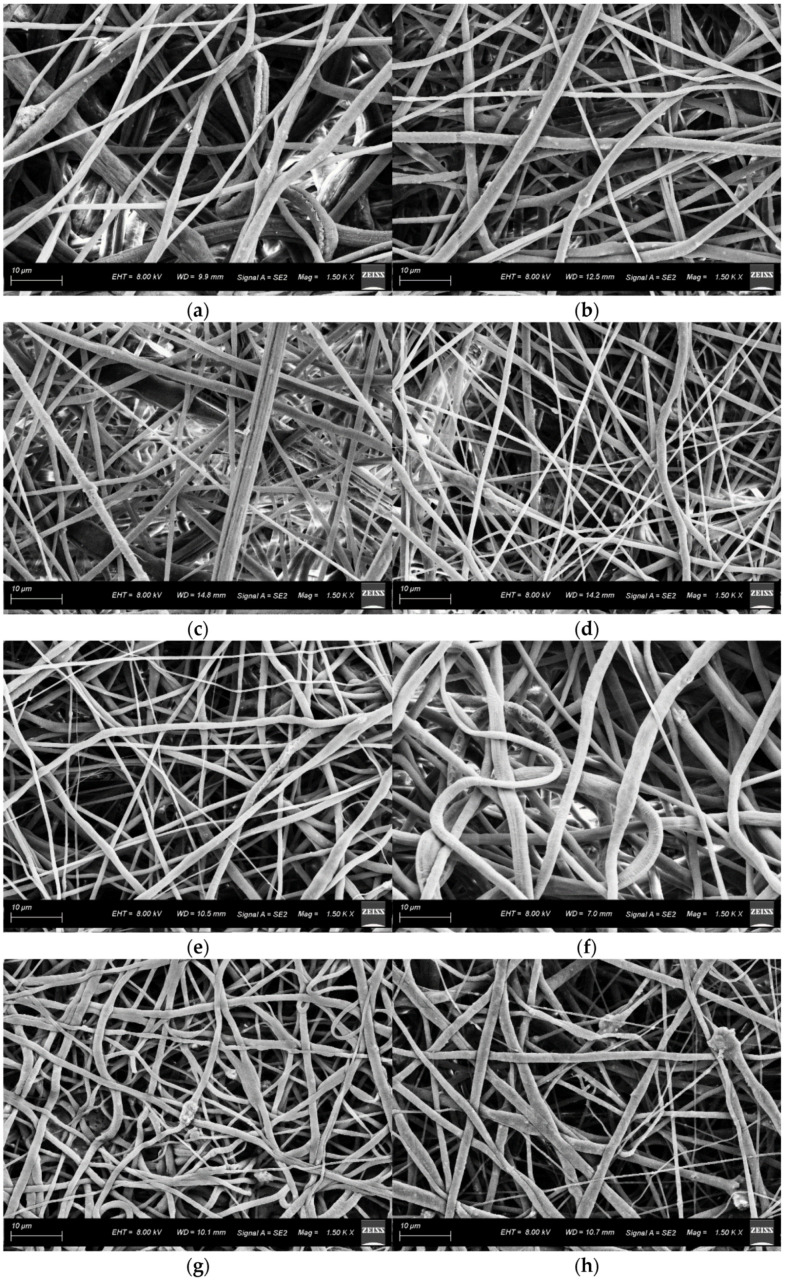

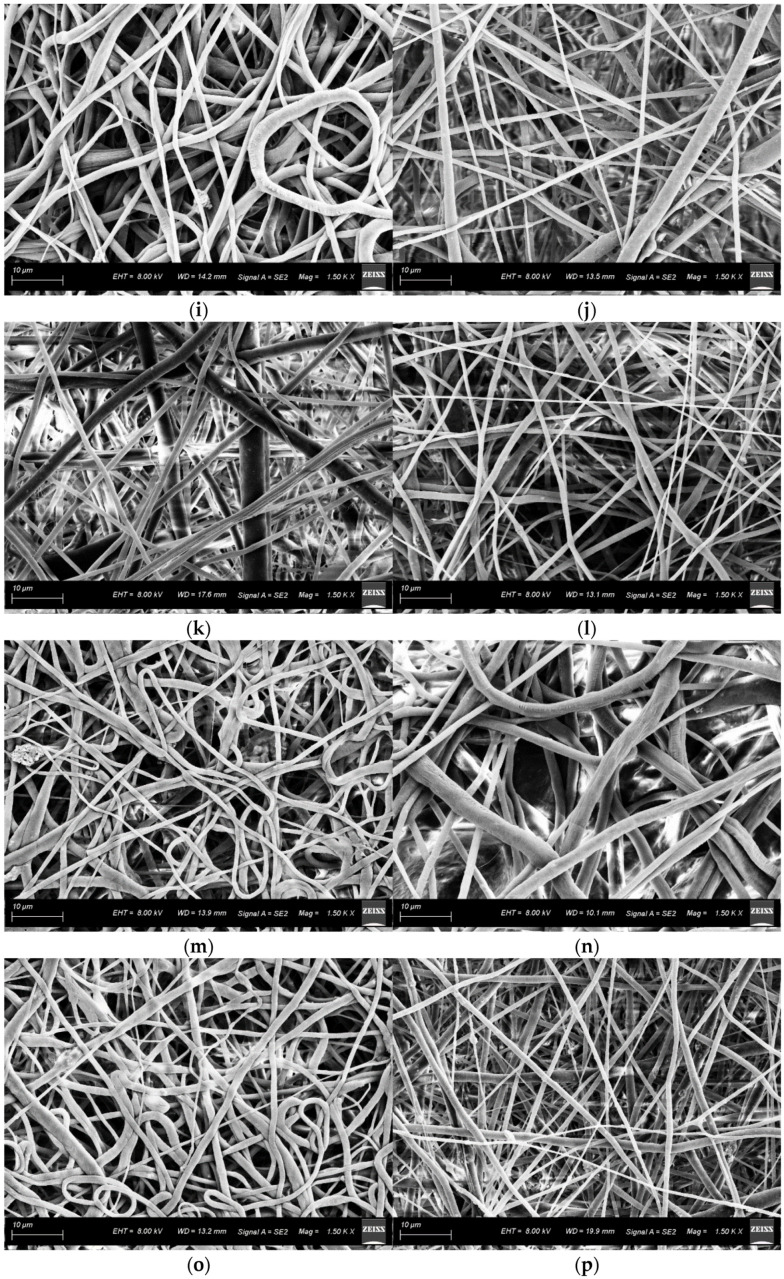
SEM micrographs of 16 specimens (**a**–**p**) prepared for Taguchi orthogonal array analysis. All the SEM micrographs were collected at 1500× magnification and at an accelerating voltage of 8 kV.

The SEM images obtained for all 16 specimens are depicted in Figure 1a–p, respectively.

The modulus, TS, and MFD values calculated for all 16 specimens were tabulated in Table 1. The MFD, modulus, and TS values of tested specimens varied from 932 to 1925 nm, 16.62 to 207.3 MPa, and 1.2 to 3.5 MPa, respectively. ANOVA outlined ESM wt%, HAp wt%, AV, FR, SCD, and an interactive effect between PCL wt% and AV to be the significant factors affecting the modulus values of an electrospun PCL/ESM membrane (*p* < 0.05). Furthermore, concentrations of PCL and ESM were the significant factors affecting MFD values (*p* < 0.05). However, there were no significant factors observed to be affecting the TS values.

**Figure 2:**
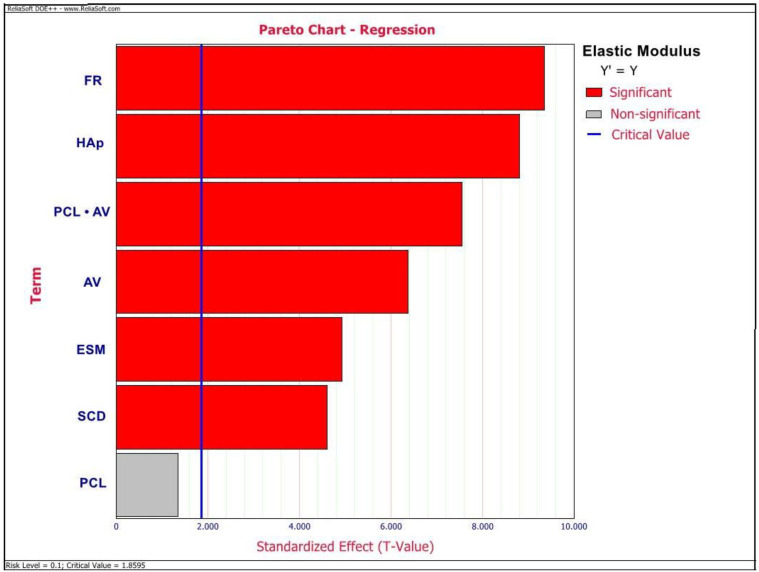
Pareto chart depicting the significant factors affecting elastic modulus values of an electrospun composite membrane.

**Figure 3:**
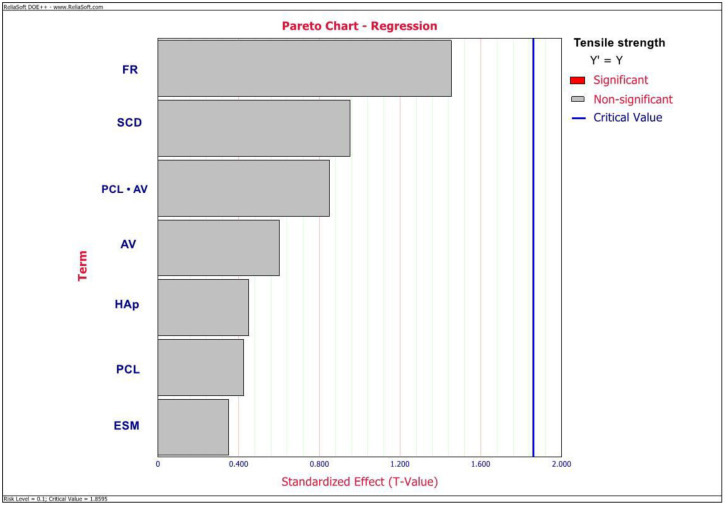
Pareto chart depicting the significant factors affecting TS values of an electrospun composite membrane.

**Figure 4:**
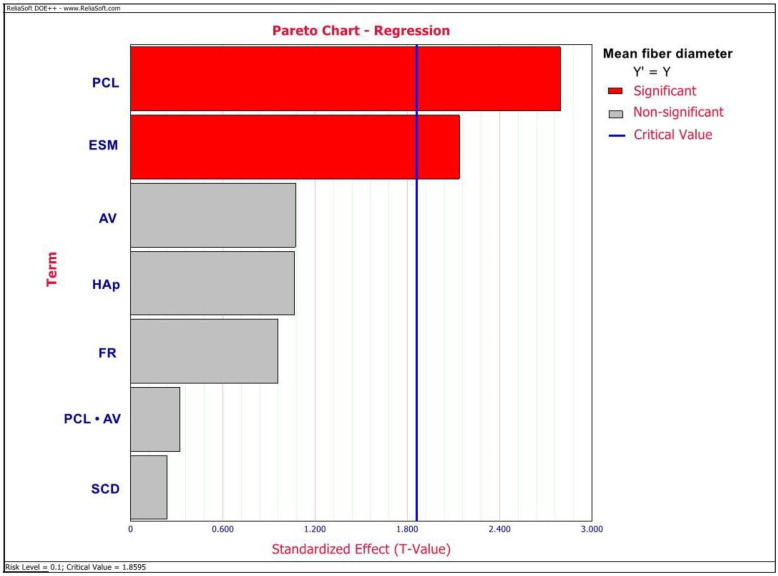
Pareto chart depicting the significant factors affecting mean MFD values of an electrospun composite membrane.

ANOVA outlined ESM wt%, HAp wt%, AV, FR, SCD, and an interactive effect between PCL wt% and AV to be the significant factors affecting the elastic modulus values of this GTR/GBR membrane (*p* < 0.05) as observed in the Pareto chart depicted in Figure 2. However, there were no significant factors observed to be affecting the TS values as depicted in the Pareto chart in Figure 3. Additionally, from the Pareto chart, as depicted in Figure 4, PCL and ESM concentrations were observed to be the significant factors affecting MFD values (*p* < 0.05).

The analytical quadratic model obtained by multiple regression analysis for variations within response variables of this GTR/GBR membrane is shown in Equation (1). The coefficients of the quadratic Equation (1) after fitting the data to analyzed response variables are tabulated in Table 2.

**Y = X + X_1_A + X_2_B + X_3_C + X_4_D + X_5_E + X_6_F + X_7_(A·D)** (1)

Y is the response variable analyzed. A, B, C, D, E, and F are the coded values for PCL wt%, ESM wt%, HAp wt%, AV, FR, and SCD, respectively. X, X_1_, X_2_, X_3_, X_4_, X_5_, X_6_, and X_7_ are the respective coefficients.

**Table 2:** Regression information for analyzed response variables.

Y	X/SE *	X_1_/SE *	X_2_/SE *	X_3_/SE *	X_4_/SE *	X_5_/SE *	X_6_/SE *	X_7_/SE *
**Elastic Modulus**	97.4075/4.8274	−9.2295/6.8269	23.8167/4.8274	42.5065/4.8274	−30.7697/4.8274	45.1215/4.8274	22.2252/4.8274	51.5422/6.8269
**Tensile Strength**	2.425/0.2494	−0.15/0.3527	−0.0875/0.2494	−0.1125/0.2494	0.15/0.2494	0.3625/0.2494	−0.2375/0.2494	0.3/0.3527
**Mean Fiber Diameter**	1313.25/54.094	213.75/76.5004	−115.625/54.094	−57.625/54.094	58 /54.094	51.875/54.094	12.875/54.094	−24.5/76.5004

* SE = standard error.

**Figure 5:**
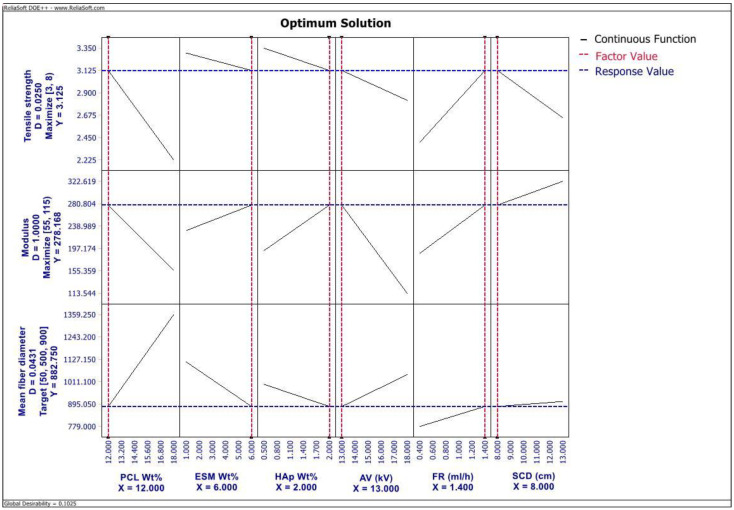
The optimal solution for maximizing the mechanical properties and MFD values of an electrospun composite membrane.

Additionally, optimization using DOE++ software predicted that the maximal TS of 3.125 MPa, modulus of 278.168 MPa, and MFD of 882.75 nm could be achieved using 12 wt% PCL, 6 wt% SEP, 2 wt% HAp, 13 kV AV, 1.4 mL/h FR, and 8 cm SCD with a global desirability of 0.1025 as depicted in Figure 5.

## 3. Discussion

For GTR/GBR membrane applications, adequate mechanical properties are a primary requisite to prevent collapse into the underlying alveolar bone defect region and to protect the bone defect region for alveolar bone and periodontal ligament regeneration. Thus, optimization of mechanical properties, by controlling various electrospinning parameters and mean fiber diameter is necessary to obtain a durable GTR/GBR membrane with maximum mechanical properties. In this study, there were six independent parameters (PCL wt%, ESM wt%, HAp wt%, AV, FR, SCD) that could alter the desired responses and each parameter had two levels (low and high). Hence, there could be 64 different combinations of parameters per response variable for the preparation of this membrane, using a full-factorial method. To avoid the cumbersome process of preparing 64 groups of specimens and to save time and resources, the method of Taguchi orthogonal arrays was used to generate a reduced set of combinations of independent factors to derive an optimum combination of the parameters necessary for the preparation of this membrane. Taguchi orthogonal array is a fractional factorial method that ensures equal consideration of all the levels of all factors and evaluates the effects of individual parameters and the lower-order two-way interactions [22]. Our experimental design strategy was based on the Pareto principle, which advocates that 20% of the main factors and their lower-order interactions account for 80% of the outcomes [23]. This also implies that the higher-order interactions typically do not have a significant effect on the response, and hence was rational to use a Taguchi orthogonal array design for this study. DOE++ generated 16 combinations of parameters and thus 16 n specimens were used for analysis.

Bead-on-string fibers were usually considered a “by-product” of the electrospun fibers and confer detrimental properties to the electrospun membrane by reducing the surface area of the membrane [24,25]. Thus, the production of smooth bead-free uniform fibers is desirable. From the SEM micrographs (Figure 1a–p), we can observe that the bead-free uniform fibers are formed for all the tested specimens. Furthermore, from the SEM images (Figure 1a–p), we can observe the presence of HAp agglomerates. The presence of agglomerates within nanofibrous scaffolds on the addition of bioceramics as HAp and β- tricalcium phosphate was also previously reported by Castro VO et al. [26] and Santos VI et al. [27].

From the results (Figure 2 and Figure 5 and Table 2), we can observe that except for AV, the rest of all significant factors, including ESM wt%, HAp wt%, SCD, FR, and interaction between PCL wt% and AV have a positive correlation with an elastic modulus of this membrane. Regarding TS of th membrane, the Pareto chart (Figure 3) showed that there are no significant parameters affecting TS. However, from the regression information (Table 2), we can observe that TS has a negative correlation with all tested parameters except for FR, AV, and the interaction between PCL wt% and AV. The results observed in this study were in contrast with the results reported by Xiong X et al. [13], Jia J et al. [14], and Qi Q et al. [28], wherein they observed increased mechanical properties of ESM-based membranes on incorporating increased amounts of the additive synthetic polymers and chitosan. This might be due to the interaction with bioceramic, HAp in this composite and could also be attributed to the difference in the processed commercially available ESM powder used in this study.

Furthermore, PCL wt% and ESM wt% are the significant factors affecting the MFD of the PCL/ESM membrane (Figure 3). Additionally, the optimal solution (Figure 5) and regression information for MFD (Table 2) demonstrated that except for ESM wt%, HAp wt%, and interaction between PCL wt% and AV, all other tested factors have a positive correlation with the MFD of final nanofibers. The negative correlation of MFD with HAp wt% observed in this study agrees with results reported by Doustgani A et al. [29], wherein they attributed it to the increase in conductivity of the polymer solution and the surface charge density of the solution jet with the addition of HAp. Furthermore, the negative correlation of MFD with ESM wt% could also be attributed to the increased surface charge density of polymer solution jet with increased ESM concentration.

Additionally, the optimization of the formulation to prepare an electrospun PCL/ESM GTR/GBR membrane with global maximal mechanical properties such as TS, and elastic modulus as well as to achieve the MFD close to the collagen fibers in the native extracellular matrix, is completed with an optimization tool in DOE++ software. Since we have three responses (TS, modulus, and MFD), the optimal setting of independent variables for one of the responses may not be good for the other two. Therefore, a compromise should be made and a balanced setting of independent variables that can optimize the overall desired responses should be found. DOE++ software used in this study employed a statistical and mathematical approach to efficiently optimize the mechanical properties including modulus and TS, along with targeted MFD values for this electrospun composite membrane by a compromise between the minimal requirements, target level, and relative importance of each desired outcome. From Figure 5, we can observe that the PCL/ESM electrospun membrane prepared using polymer solution concentrations of 12 wt% of PCL, 6 wt% of ESM, 2 wt% of HAp, and electrospinning parameters of 13 kV AV, 1.4 mL/h FR, and 8 cm SCD will have the predicted TS of 3.125 MPa with a desirability value of 0.025; predicted modulus of about 278.168 MPa with a desirability value of 1; and a predicted MFD of 882.75 nm with a desirability value of 0.0431. The achieved overall desirability value is 0.1025.

Apart from the parameters tested in this study, the effect of additional environmental parameters such as temperature, relative humidity, and additional polymer solution parameters such as viscosity and solution conductivity could be investigated further. Future studies correlating the effect of mechanical properties and MFD with degradation properties are necessary to determine the applicability of these nanofibers in various biomedical applications. Additionally, characterization of the optimized GTR/GBR membrane to determine the porosity, chemical structure, physical properties, and biocompatibility testing using cell culture studies are necessary to validate the applicability of these nanofibrous GTR/GBR membranes in periodontal regeneration applications.

## 4. Materials and Methods

Poly (ε-caprolactone) (PCL, Mn:80,000), Chloroform (CF, ≥99.5%), and N, N-Dimethylformamide (DMF, 99.8%) were procured from Sigma-Aldrich (Millipore Sigma, St. Louis, MO, USA). Nano-Hydroxyapatite (HAp, particle size:100 nm) was purchased from Berkeley advanced biomaterials (Berkeley, CA, USA). Natural eggshell membrane powder was bought from Healthy Origins (Pittsburg, PA, USA). All reagents were used as received without further purification.

### 4.1. Taguchi Orthogonal Array Study Design

In this study, the independent variables such as PCL wt%, ESM wt%, HAp wt%, AV, FR, and SCD used for the preparation of electrospun membrane were considered as input factors while, TS, elastic modulus, and MFD were measured as the three objectives or response variables. A Taguchi orthogonal array L8 design (DOE folio, Weibull++ software, Reliasoft, AZ, USA) using two levels of factors (low and high) was used to screen out significant factors affecting the MFD and mechanical properties of this electrospun membrane as well as to obtain an optimum formulation of independent variables that could maximize the modulus, TS, and MFD values. The range and levels of the independent factors which were used in optimization are tabulated in Table 3. The minimum and maximum levels of the independent factors were chosen by considering the preliminary studies, trial experiments, practical limitations, and represent the attainable limits for nanofiber formation and/or equipment operation. For instance, the polymer solution becomes highly viscous and could not be electrospun at PCL concentrations greater than 18 wt%, particularly when used together with ESM concentrations greater than 6 wt%. Furthermore, HAp concentrations greater than 2 wt% resulted in agglomerate formation and precipitation of HAp powder particles at the bottom of the test tube during polymer solution preparation. At PCL concentrations lesser than 12 wt%, there are beads and spindle-shaped fiber formation observed within the electrospun fiber mats, even with higher ESM concentrations.

Based on the range of input variables, no. of replicates, and desired response variables, Taguchi orthogonal array design folio in DOE++ software generated 16 electrospun membrane specimen combinations (two replicates of eight combinations of various independent parameters (2n × 8 combinations)) with varying concentrations of PCL, ESM, HAp, and altered electrospinning parameters needed to run the analysis.

### 4.2. Preparation of Electrospun Membrane Specimens

The 16 specimen combinations generated by DOE++ software as tabulated in Table 4 were prepared using the blend electrospinning technique. Polymer solution for the blend electrospinning process was prepared by initially dissolving the premeasured quantity of HAp powder (Table 4) in a mixture of CF and DMF (3:1) solvents for 30 min using a sonicator. Then, premeasured quantities of PCL and ESM as tabulated in Table 4 were blended and dissolved in the HAp/CF/DMF mixture overnight using a bench rocker. The resulting PCL/ESM solution was loaded into a syringe and an 18-gauge needle is attached to it to carry out the electrospinning process using a custom electrospinning setup [30]. The same process was repeated with various polymer solution concentrations and altered electrospinning parameters for the preparation of 16 specimens as tabulated in Table 4. All 16 specimens were collected after 10 h of electrospinning to obtain the desired thickness range at the laboratory conditions of 23 ± 2 °C temperature and 50% ± 1% relative humidity and left to dry overnight before analysis to allow for the residual solvent evaporation.

### 4.3. Mechanical Testing and MFD Assessment of PCL/ESM Membrane Specimens

The morphology of all the fibrous mats was characterized using Field-Emission Scanning Electron Microscopy (SEM, Supra 40, Carl Zeiss, Germany). Specimens were sputter-coated with Ag-Pd alloy using a sputter coater (Q150T, Carl Zeiss, Germany) to improve the electrical conductivity, then observed using SEM at a voltage of 8 kV and 1500 X magnification. ImageJ software (ImageJ, Version 1.53b, National Institute of Health, Bethesda, MD, USA) was used to assess the MFD based on the obtained SEM images. Mean fiber diameter was calculated as an average of fiber diameters measured at 30 different positions on each SEM image of the specimen.

**Figure 6:**
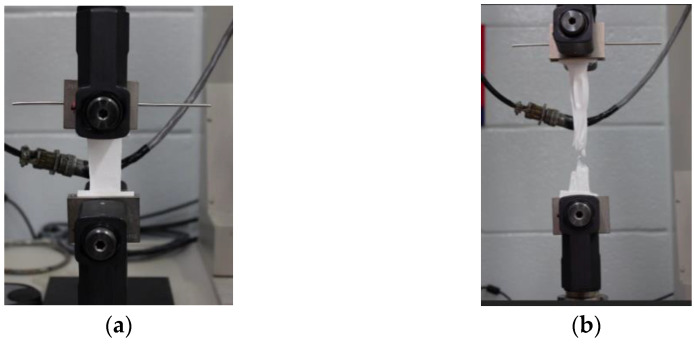
Electrospun membrane specimen (**a**) loaded onto the Sintech 2/G materials testing system for uniaxial tensile testing, and (**b**) tested till failure.

For the mechanical testing, all specimens were prepared to have a width of 13 mm and further taped at both ends to prevent gripping failures. The VHX Digital Microscope (Keyence Corp, Osaka, Japan) was used to precisely record the thickness (*n* ≥ 5) of the electrospun membrane without any compression by mechanical instrumentation. Uniaxial tensile testing was carried out according to ASTM D882 Standard test method using a Sintech 2/G Materials Testing System (MTS, Eden Prairie, MN, USA) at an extension rate of 0.5 in/min as depicted in Figure 6a,b. Mechanical properties such as elastic modulus and TS were calculated using MTS Testworks 4.0 software (MTS, Eden Prairie, MN, USA).

### 4.4. Screening and Optimization of Significant Factors

After the determination of MFD, TS, and modulus values for all 16 specimens, the obtained values were input into DOE++ software and Taguchi orthogonal array analysis was run. From the Taguchi analysis, ANOVA was used to screen the independent parameters that had significant effects on the MFD, TS, and elastic modulus of the final membrane (α = 0.05). Furthermore, after finding the significant factors and interactions, optimization was completed using the optimize feature in the DOE++ software to find the optimum values of independent factors needed to maximize modulus and TS as well as to achieve a maximum MFD of around 500 nm to resemble the MFD values of collagen fibers in the natural extracellular matrix as reported in the literature [31,32].

## 5. Conclusions

To conclude, the results showed that the elastic modulus of this novel PCL-based GTR/GBR membrane varies with the ESM wt%, HAp wt%, AV, FR, SCD, and an interactive effect between PCL wt% and AV. However, there is no significant variation observed with TS values with any of the parameters tested. Additionally, there is a significant variation in MFD values observed with ESM and PCL polymer concentrations within the electrospinning polymer solution. Thus, the null hypothesis stating that there was no significant variation in mechanical properties and MFD of final membranes with altered electrospinning parameters and polymer solution concentration was partially accepted.

Furthermore, the results show that the Taguchi orthogonal arrays and optimization by Design of Experiments employed in this study are effective modeling tools for screening and optimizing various parameters during the preparation of this novel GTR/GBR membrane.

## Figures and Tables

**Table 1:** TS, modulus, and MFD values for all 16 specimens.

n #	Tensile Strength (MPa)	Elastic Modulus (MPa)	Mean Fiber Diameter (nm)
1	3.5	128.678	1649
2	2.3	194.813	1459
3	1.7	31.578	1201
4	2.6	29.981	932
5	2.9	68.888	993
6	3.1	104.391	1925
7	3	32.196	1067
8	1.9	23.488	1261
9	1.3	89.305	1532
10	1.2	207.338	1311
11	0.8	20.061	1592
12	1.6	16.622	956
13	2.7	41.67	1258
14	3	115.557	1672
15	3.1	24.867	1259
16	1.7	16.749	1141

**Table 3:** The range and levels of the independent variables used for Taguchi orthogonal array design.

Independent Variables	Level 1 (Low)	Level 2 (High)
PCL wt%	12	18
ESM wt%	1	6
HAp wt%	0.5	2
Applied Voltage (AV) (kV)	13	18
Flow Rate of Solution (FR) (mL/h)	0.4	1.4
Spinneret to Collector Distance (SCD) (cm)	8	13

**Table 4:** The 16 specimen combinations of various input independent variables generated by DOE++.

n #	PCL (wt%)	SEP (wt%)	HAp (wt%)	Applied Voltage (kV)	Flow Rate (ml/h)	S-C Distance (cm)
1	18	1	2	13	1.4	8
2	18	6	2	13	1.4	13
3	18	6	0.5	13	0.4	13
4	12	6	2	18	0.4	8
5	12	1	0.5	13	0.4	8
6	18	1	0.5	18	1.4	13
7	12	6	0.5	18	1.4	8
8	12	1	2	18	0.4	13
9	18	1	2	13	1.4	8
10	18	6	2	13	1.4	13
11	18	6	0.5	13	0.4	13
12	12	6	2	18	0.4	8
13	12	1	0.5	13	0.4	8
14	18	1	0.5	18	1.4	13
15	12	6	0.5	18	1.4	8
16	12	1	2	18	0.4	13

## Data Availability

Not applicable.
